# Comparison of Real-Time Quantitative PCR and Culture for the Diagnosis of Emerging Rickettsioses

**DOI:** 10.1371/journal.pntd.0001540

**Published:** 2012-03-06

**Authors:** Emmanouil Angelakis, Hervé Richet, Jean-Marc Rolain, Bernard La Scola, Didier Raoult

**Affiliations:** URMITE UMR 6236, CNRS-IRD, Faculté de Médecine et de Pharmacie, Marseille, France; University of Texas Medical Branch, United States of America

## Abstract

**Background:**

Isolation of *Rickettsia* species from skin biopsies may be replaced by PCR. We evaluated culture sensitivity compared to PCR based on sampling delay and previous antibiotic treatment.

**Methodology/Principal Findings:**

Skin biopsies and ticks from patients with suspected *Rickettsia* infection were screened for *Rickettsia* spp. using qPCR, and positive results were amplified and sequenced for the *gltA* and *ompA* genes. Immunofluorescence for spotted fever group rickettsial antigens was done for 79 patients. All skin biopsies and only ticks that tested positive using qPCR were cultured in human embryonic lung (HEL) fibroblasts using the centrifugation-shell vial technique. Patients and ticks were classified as definitely having rickettsioses if there was direct evidence of infection with a *Rickettsia* sp. using culture or molecular assays or in patients if serology was positive. Data on previous antibiotic treatments were obtained for patients with rickettsiosis. *Rickettsia* spp. infection was diagnosed in 47 out of 145 patients (32%), 41 by PCR and 12 by culture, whereas 3 isolates were obtained from PCR negative biopsies. For 3 of the patients serology was positive although PCR and culture were negative. *Rickettsia africae* was the most common detected species (n = 25, [17.2%]) and isolated bacterium (n = 5, [3.4%]). The probability of isolating *Rickettsia* spp. was 12 times higher in untreated patients and 5.4 times higher in patients from our hometown. *Rickettsia* spp. was amplified in 24 out of 95 ticks (25%) and we isolated 7 *R. slovaca* and 1 *R. raoultii* from *Dermacentor marginatus*.

**Conclusions/Significance:**

We found a positive correlation between the bacteria copies and the isolation success in skin biopsies and ticks. Culture remains critical for strain analysis but is less sensitive than serology and PCR for the diagnosis of a *Rickettsia* infection.

## Author Summary

Diagnosis of *Rickettsia* infection would benefit by use of the more rapid and sensitive method of quantitative real-time PCR than the time-intensive and less sensitive method of culturing *Rickettsia* species from skin biopsies. We evaluated culture sensitivity compared to PCR according to sampling delay and previous antibiotic treatment. We found that skin biopsies can be positive even when molecular tests were negative, and a negative result using molecular assays did not exclude the diagnosis of *Rickettsia* spp. infection. *Rickettsia africae* was the most common species in skin biopsies and *R. slovaca* was most common in ticks. We found a positive correlation between the number of bacteria copies and the isolation success in skin biopsies and ticks. The probability of isolating *Rickettsia* spp. was higher in untreated patients and in patients from our hometown. To increase the sensitivity of culture, skin biopsies should be sampled before treatment early in the course of the disease and should be inoculated as soon as possible.

## Introduction

Rickettsial diseases are zoonoses caused by obligate intracellular bacteria found in the order Rickettsiales [1]. In the past, only research laboratories were able to isolate rickettsiae from clinical specimens [2]. However, in recent years, the development of cell culture systems for viral isolation has led to an increase in the number of laboratories suitably equipped to isolate rickettsiae [2]. The isolation of *Rickettsia* species from samples using cell culture (especially the shell vial technique) remains critical for the description of new species, enabling genetic descriptions, physiological analyses, improvement in diagnostic tools, and antibiotic susceptibility testing of bacteria [2]. The isolation of rickettsial organisms is often difficult, and the success of culturing *Rickettsia* spp. is based on the numbers of microorganisms in cells (which should be as high as possible) and on the centrifugation step, which enhances the adhesion of bacteria that are freed from their intracellular location to the cells in culture [3], [4]. Moreover, early antibiotic treatment prior to the biopsy has been significantly associated with a reduced culture efficacy [5]. To reduce the delay in diagnosis, quantitative real-time PCR (qPCR) for the diagnosis of human rickettsiosis allows for both convenient and rapid detection and the identification of rickettsiae [6]. As a national reference center for rickettsioses, we routinely receive specimens from patients with suspected *Rickettsia* infections. In this study, we analyzed a large collection of skin biopsies and ticks collected from patients with suspected *Rickettsia* infections using molecular techniques and shell vial cell cultures. Our objective was to evaluate cell culture techniques useful for the diagnosis of *Rickettsia* infections in comparison with PCR.

## Materials and Methods

### Samples

We studied punch biopsies or scalpel incisions of eschars and ticks collected from patients with suspected rickettsial infections between January 2007 and January 2010. For some patients a serum sample was also collected. Specimens sent to our reference center were obtained from both hospitalized patients and outpatients throughout France. Skin biopsies and ticks were sent either frozen or in transport media whereas serum samples were sent in room temperature. Skin biopsies and ticks were screened for the presence of *Rickettsia* spp. using qPCR, and for positive results PCR amplification and sequencing were used for the identification of *Rickettsia* at the species level. Only ticks that tested positive were identified at the species level. We cultured all skin biopsies and only ticks that were PCR positive. Patients were classified as definitely having rickettsioses if there was direct evidence of infection with a *Rickettsia* sp. using culture or molecular assays or if serology was positive. Ticks were classified as definitely having rickettsioses if culture or molecular assays were positive. Data on previous antibiotic treatments were obtained for patients with rickettsioses.

### Molecular methods

Total genomic DNA was extracted from samples using a QIAamp tissue kit (Qiagen, Hilden, Germany). Samples were handled under sterile conditions to avoid cross-contamination. Genomic DNA was stored at 4°C as used as a template in PCR assays. Samples were screened for the presence of *Rickettsia* spp. using a previously developed qPCR assay targeting a 109-bp fragment of a hypothetical protein as previously described [7]. If a positive result was obtained, PCR amplification and sequencing targeting the *gltA* and *ompA* genes were used, as previously described [8]. A maximum of 10 samples were tested along with negative controls (DNA from uninfected skin biopsies or ticks and sterile water) and a positive control (DNA from *Rickettsia montanensis*). Tick DNA was also used as a template in a previously described qPCR assay targeting *Dermacentor* 12 S rRNA to identify the ticks [9]. The quality of DNA handling and extraction of human samples was verified by qPCR for a housekeeping gene encoding beta-actin [10].

### Quantification of *Rickettsia* spp

**Figure 1:**
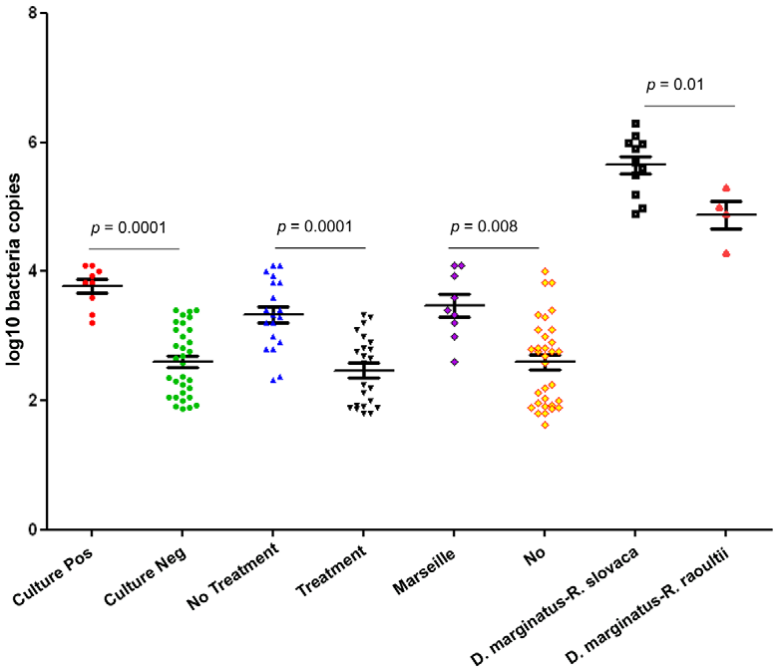
Comparison between the cycles and log10 values of the number of hypothetical protein copies/ml for *R. africae*.

A previous described gene [7] was used for the quantification of *Rickettsia* spp.. Serial ten-fold dilutions (from 10^−1^ to 10^−11^) of *R. africae*, *R. slovaca*, *R. raoultii*, and *R. helvetica* were performed. Bacteria were detected by indirect immunofluorescence using human serum and antiserum. The number of copies/ml was calculated from the highest dilution down to the dilution that contained at least one bacterium, corresponding to 10^4^ bacteria/ml. Each dilution was tested using hypothetical protein qPCR to express Ct in terms of the number of bacteria/ml and copies of qPCR/ml per sample [11] (Figure 1).

### Culture methods

Samples were cultured in human embryonic lung (HEL) fibroblasts using the centrifugation-shell vial technique (Sterilin-Felthan-England, 3.7 ml) using 12-mm round coverslips seeded with 1 ml of medium containing 50,000 cells and incubated in a 5% CO_2_ incubator at 37°C for three days to obtain a confluent monolayer [4], [12]. Cultures were surveyed for four weeks, and bacterial growth was assessed every seven days on cover slips directly inside the shell vial using Gimenez and immunofluorescence staining [4], [12]. When the staining method was positive, the *Rickettsia* isolate was identified using PCR and sequencing as described above [8].

### Serology

All sera were tested by immunofluorescence (IF) for spotted fever group (SFG) rickettsial antigens (*R. conorii conorii*, *R. india*, *R. japonica*, *R. felis*, *R. honei* and *R. heilongjiangensis*) as previously described [13]. IF was considered positive for *Rickettsia* spp. infection when there was a four-fold rise in the antibody titer or a single antibody titer of IgG ≥1/128 combined with an IgM titer ≥1/64 against one or more antigens of the tested species [13].

### Statistical analysis

For data comparison, the Student's *t-*test or χ^2^ test was performed using EpiInfo version 6.0 software (Centers for Disease Control and Prevention, Atlanta, GA, USA). A *p* value<0.05 was considered significant. In addition, a principal component analysis was performed using PASW Statistics 17.0 software (Chicago, Illinois, USA) to assess the correlation between the following variables: molecular assay results, culture results, previous patient treatment, and patient locality (from Marseille or elsewhere). The results of the analysis are shown on factor loading plots. To assess which factor had the greatest importance for the isolation of *Rickettsia* spp., a binary logistic regression was performed using PASW Statistics.

### Ethic statement

This study is based on routine diagnosis samples, all collected within the Rickettsioses National Reference Center mission.

## Results

### Diagnoses in patients

We tested 145 skin biopsies from patients with suspected rickettsiosis and *Rickettsia* spp. infection was diagnosed in 47 (32%) (Table 1). Twenty three (48%) patients had already an antibiotic treatment when the skin biopsy was sampled. By qPCR a positive result was obtained for 41 skin biopsies (28.2%). *Rickettsia africae* was the most common detected species (n = 25, [17.2%]) followed by *Rickettsia conorii conorii* (n = 7), *Rickettsia slovaca* (n = 4), *Rickettsia sibirica mongolitimonae* (n = 4) and *Rickettsia raoultii* (n = 1) (Table 1). *Rickettsia* spp. were isolated from 12 skin biopsies (8%). Eleven isolates (91%) were from untreated patients and only 1 isolate (*R. conorii conorii*) from a patient who had already had a single dose of doxycycline (100 mg) about 8 hours before. Three isolates were from biopsies that were negative using qPCR. The beta-actin gene expression for these three skin biopsies was strongly positive showing a good DNA extraction procedure. Overall, 8 skin biopsies (19%) were positive by PCR were also positive by isolation (mean ± standard error of the mean (SEM) cycles (Ct) values) (25.3±0.1). *R. africae* was the most commonly isolated bacterium (n = 5, [3.4%]) followed by *R. conorii conorii* (n = 3), *R. sibirica mongolitimonae* (n = 2) and *R. slovaca* (n = 2).

**Table 1:** Results of PCR assay, culture and serology for the 145 patients tested.

Diagnosis of rickettsial infection	*Rickettsia*-positive PCR	Culture positive	Serology		Total
			Acute sample	Convalescent-phase sample	
*Rickettsia africae*	25	5	0	11	26
*Rickettsia conorii conorii*	7	3	3	4	10
*Rickettsia slovaca*	4	2	0	2	6
*Rickettsia sibirica mongolitimonae*	4	2	0	0	4
*Rickettsia raoultii*	1	0	0	0	1
Total	41	12	3	17	47

For 79 patients with suspected rickettsiosis we received a serum sample. For 53 patients we only received an acute serum sample whereas for 26 patients we both received an acute and a convalescent-phase serum sample. We found 3 (3.7%) acute serum samples and 17 (65%) convalescent-phase serum samples positive by IFA (Table 1). For 3 patients serology was positive although PCR and culture were negative.

### Diagnoses in ticks

We tested 95 ticks removed from 95 patients, and 24 (25%) were positive. *R. slovaca* was the most frequently amplified *Rickettsia* sp. (n = 11; 12%). All *R. slovaca* specimens were amplified from *D. marginatus* ticks. We also amplified *R. raoultii* from *D. marginatus* (n = 4), *Rickettsia helvetica* from *Ixodes ricinus* (n = 4), *Rickettsia massiliae* from *Rhipicephalus sanguineus* (n = 3), *R. conorii conorii* from *R. sanguineus* (n = 1) and *R. africae* from *Amblyomma variegatum* (n = 1; Table 2). A total of 24 positive ticks were cultured, and isolates were obtained from 8 (34%). *R. slovaca* was the most commonly isolated bacterium (n = 7), and we also isolated one *R. helvetica* specimen. For 29 patients we received a serum sample. Eighteen patients had only an acute serum and 11 patients had both acute and convalescent-phase serum samples. No acute serum samples and 7 (63%) convalescent-phase serum samples were positive by IFA. All 7 positive sera were from patients who had a *Rickettsia* sp. positive tick.

**Table 2:** Results of PCR assays and culture of the 95 ticks.

Tick species	*Rickettsia* spp. infection (Number)	Culture positive	%
*Dermacentor marginatus*	*Rickettsia slovaca* (11)	7	64%
	*Rickettsia raoultii* (4)	-	0%
*Ixodes ricinus*	*Rickettsia helvetica* (4)	1	25%
*Rhipicephalus sanguineus*	*Rickettsia massiliae* (3)	-	0%
	*Rickettsia conorii conorii* (1)	-	0%
*Amblyomma variegatum*	*Rickettsia africae* (1)	-	0%
Total	24	8	33%

### Comparison of qPCR results in humans and ticks

**Figure 2:**
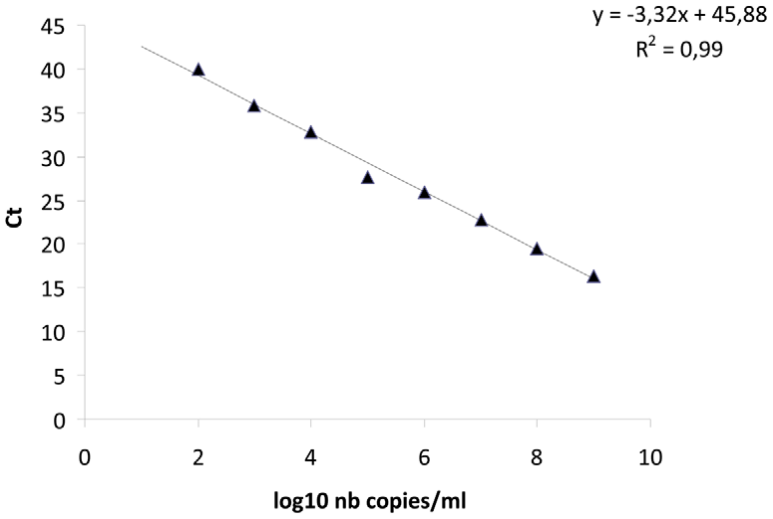
The mean ± standard error of the mean (SEM) of log10 copies obtained using qPCR. Culture pos: culture positive skin biopsies, Culture neg: culture negative skin biopsies; No treatment: skin biopsies obtained from patients without treatment, Treatment: skin biopsies obtained from patients receiving a treatment; Marseille: skin biopsies obtained from patients from Marseille, No: skin biopsies obtained from patients from elsewhere; *D. marginatus-R. slovaca*: *D. marginatus* ticks infected by *R. slovaca*, *D. marginatus-R. raoultii*: *D. marginatus* ticks infected by *R. slovaca*.

The mean ± SEM copies obtained using qPCR revealed that culture-positive samples presented significantly higher copies than culture-negative biopsies (3.7±0.1 versus 2.6±0.09 respectively; *p* = 0.0001 using Student's *t-*test) (Figure 2). No difference in the Ct values for beta actin found between culture positive and culture negative samples (25.5±0.6 versus 26.1±0.3 respectively; *p* = 0.5 using Student's *t-*test). Biopsies from untreated patients presented significantly higher copies than those from treated patients (3.3±0.1 versus 2.4±0.1, respectively; *p* = 0.0001 using Student's *t-*test). No difference in the Ct values for beta actin found between the skin biopsies of treated and untreated patients (24.7±1.1 versus 25.1±0.6 respectively; *p* = 0.6 using Student's *t-*test). Biopsies from Marseille presented significantly higher copies than elsewhere (3.5±0.2 versus 2.6±0.1, respectively; *p* = 0.008 using Student's *t-*test) and no difference in the Ct values for beta actin (25.7±0.3 versus 25.1±0.5 respectively; *p* = 0.7 using Student's *t-*test). In addition, *D. marginatus* ticks infected by *R. slovaca* presented significantly higher copies than *D. marginatus* ticks infected by *R. raoultii* (5.6±0.1 versus 4.8±0.3, respectively; *p* = 0.01 using Student's *t-*test) (Figure 2).

### Comparison of culture and qPCR to serology

Comparison of culture and qPCR to serology was done for the 26 patients with suspected rickettsiosis with a skin biopsy that also had an acute serum and a convalescent-phase serum sample (Table 3). qPCR sensitivity was 82% as compared to serology whereas culture sensitivity was 29.4% as compared to serology.

**Table 3:** Results for the 26 patients with suspected rickettsiosis who had an acute and a convalescent-phase serum sample.

	Positive Serology		*Rickettsia*-positive PCR	Culture positive	Total positive
	Acute sample	Convalescent-phase sample			
Patients with skin biopsies	2 (7%)	17 (65%)	14 (53%)	5 (19%)	17 (65%)

### Comparison of patient groups

Culture sensitivity was 29.2% compared to qPCR; instead for treated patients the sensitivity was 4.3% (1/23) whereas for untreated patients the sensitivity was 52% (11/21). The probability of isolating a *Rickettsia* sp. was 12.05 times higher (95% confidence interval [CI]: 1.7 to 85.5) in untreated (n = 11) than in treated patients (n = 1), (Table 4). The probability of isolating a *Rickettsia* sp. was 5.4 times higher (95% CI: 1.9 to 15.2) for patients from Marseille (n = 6 out of 24) than elsewhere (n = 6 out of 123), and in the correlogram plot, patients from Marseille and the culture-positive group were in the same component. The probability of a patient being treated before the skin biopsy was taken was 3.09-times higher for patients from elsewhere (21 out of 38) than for patients from Marseille (2 out of 9; 95% CI: 0.87 to 10.97). In the correlogram plot, skin biopsies that tested positive using molecular assays and patients who were treated and lived outside Marseille were in the same component area.

**Table 4:** Comparison of data between positive and negative cultures.

	Culture positive	Culture negative	Odds ratio	95% confidence interval	*p*
PCR-positive skin biopsy	9 (75%)	32 (31.6%)	7.610	2.167 to 26.72	0.0006
Patients without treatment	11 (91%)	10 (31%)	12.05	1.69 to 85.55	0.0005
Patients from Marseille	6 (50%)	18 (14.6%)	5.37	1.89 to 15.2	0.004
PCR-positive for ticks	8 (100%)	16 (22%)			

To assess whether or not treatment or a specimen obtained from Marseille was independently associated with positive culture, we performed a binary logistic regression using culture as the independent variable and previous treatment and specimens obtained from Marseille as dependent variables. This analysis showed that previous treatment was independently associated with negative culture, with a probability of having a positive culture of 0.62 (*p* = 0.015). In contrast, specimens obtained from Marseille were not significantly associated with a positive culture (odds ratio = 4.2, *p* = 0.1).

### Patients with both a skin biopsy and a tick sample

We received both a skin biopsy and a tick sample from six patients (4%). For all these patients we both received an acute and a convalescent-phase serum sample. All acute phase sera were negative and 2 convalescent-phase sera were positive by IFA. For two patients, both their skin biopsies and the ticks were found to be infected by *R. slovaca*. Moreover their convalescent-phase serum samples were also positive. One patient had a tick infected by *R. slovaca*, but his skin biopsy was negative. We cultured all of the skin biopsies and the three positive ticks. We isolated *R. slovaca* from one tick, but all skin biopsy cultures were negative. All patients had already started an antibiotic treatment when their skin biopsies were sampled.

## Discussion

We identified the presence of *Rickettsia* spp. in skin biopsies and ticks removed from patients using molecular methods and cell culture assays. Our qPCR assay was sensitive and versatile and has previously been evaluated for the detection of *Rickettsia* spp. [7]. Since we did not find a significant difference between the Ct values of the beta-actin gene we believe that DNA content after DNA extraction procedure was similar in all skin biopsies specimens. Furthermore, we routinely included large numbers of negative controls in our assays that were processed identically to the test samples. Moreover, the shell vial culture assay has been performed routinely on skin biopsies in our laboratory for approximately 20 years [4], and during the 3 years of the experiment, we had no contamination problems.

Culture methods were less sensitive than molecular assays for the detection of *Rickettsia* spp. Culture sensitivity was low in patients receiving antibiotic treatment because of the high susceptibility of *Rickettsia* spp. to antimicrobial agents [14]. In our series, previous antibiotic treatment significantly reduced the number of *Rickettsia* spp. found in skin biopsies. Early antibiotic treatment, prior to the skin biopsy, was also significantly associated with decreased sensitivity of PCR, which is probably linked to the decreased numbers of bacteria at the inoculation site [15]. In previous studies, we isolated *Rickettsia* spp. in 20 (9.2%) out of 217 skin biopsies obtained from patients suspected of having a rickettsial disease [12] and in 32 out of 103 (31.0%) skin biopsies from patients with definite rickettsiosis [15]. In this study, we proved that success rate can be much better (52%) if skin biopsies are obtained from patients without treatment.

The diagnosis of rickettsial infections has been characterized as a challenge because many physicians are unfamiliar with the nonspecific symptoms found during the early stages of illness [16]. Serological tests are the easiest methods for the diagnosis of rickettsiosis but seroconversion is usually detected 7–15 days after disease onset (25–28 days for *R. africae* infection) [17]. On the other hand, *Rickettsia* may be detectable in culture as early as 48–72 hours post-inoculation [18]. In this study we found that only 3 acute serum samples were positive the time the skin biopsies were sampled. To be suitable for culture, samples must be collected prior to the initiation of an antibiotic regimen and as early as possible in the course of the disease [5]. In this series of skin biopsies, we found that a previous treatment was the most critical factor associated with a negative culture. In Marseille, physicians are familiar with rickettsial infections, and samples are collected as early as possible prior to antibiotic treatment. As a result, we obtained more positive cultures from Marseille because significantly fewer patients had received antibiotic treatment when their skin was sampled. Moreover, specimens were sent to our reference center immediately after collection, and the samples were inoculated onto shell vials with minimal delay [5].

The 63% out of 11 patients who had a tick positive for *Rickettsia* sp. also presented a convalescent-phase serum sample positive for *Rickettsia* sp. several days after. However this is very small number of cases to conclude that a tick on a patient could predict whether the patient would become infected with a species within the tick. We found that *R. slovaca* was the most common *Rickettsia* sp. in ticks removed from patients, and it was the only species isolated from *D. marginatus* ticks. Sarih *et al*. found that in domestic animals from northeastern Morocco, more *D. marginatus* ticks were infected by *R. slovaca* than by *R. raoultii* [19]. Moreover, it is difficult to isolate the microorganism, and the culture of ticks positive for *R. raoultii* usually remains negative [20]. Because the success of culture usually depends on the quantity of the pathogen [3], we believe that the higher inocula of *R. slovaca* than *R. raoultii* in *D. marginatus*, described here for the first time, may contribute to the fact that *R. slovaca* was more successfully isolated from these ticks.

In conclusion, for the diagnosis of *Rickettsia* infection except serology we also used molecular and culture diagnostic tools which decreased the time of diagnosis and increased the sensitivity. However a negative result using molecular assays does not exclude the diagnosis of *Rickettsia* infection. To increase the sensitivity of culture, skin biopsies should be sampled before treatment early in the course of the disease and should be inoculated as soon as possible.
